# Investigating the Effect of Internal Bleaching With 10% Carbamide Peroxide on the Color Change of Three Types of Common Endodontic Sealers

**DOI:** 10.7759/cureus.53116

**Published:** 2024-01-28

**Authors:** M Daneshpooy, F Pournaqi Azar, F Nouri, Reza Safaralizadeh

**Affiliations:** 1 Department of Operative Dentistry, Dental Faculty, Tabriz University of Medical Sciences, Tabriz, IRN; 2 Dental and Periodontal Research Center, Dental Faculty, Tabriz University of Medical Sciences, Tabriz, IRN

**Keywords:** endofill, spectrophotometer, internal bleaching, sealer, color change

## Abstract

Background and purpose

Color change caused by materials used for endodontic treatment is an important clinical issue. The current research examined the impact of internal bleaching with 10% carbamide peroxide on discolored teeth resulting from various types of sealers.

Materials and methods

In this study, 36 anterior teeth were cut from 1 mm beneath the cementoenamel junction (CEJ), and the samples were divided into three groups of 12. Then, AH26 (Tulsa Dental, Tulsa, OK), Endofill (Herpo Produtos Dentários Ltda, Petrópolis, Brazil), and AH Plus (Dentsply DeTrey, Konstanz, Germany) color change potential sealers were placed inside the pulp chamber. The cervical access cavity was covered with a thin layer of glass ionomer. After one month, the material was removed, and bleaching was done with 10% carbamide peroxide. The color of the samples was measured by a spectrophotometer before bleaching, one week after bleaching, and one week after bleaching again. The data were subjected to statistical analysis using the Statistical Package for Social Sciences (SPSS) software version 16 (IBM SPSS Statistics, Armonk, NY), with a significance level set at P<0.05.

Results

The results showed that the factor of time and material used and the opposing effect of these two on the amount of L and ΔE were statistically significant (P<0.05). After one to two weeks of internal bleaching, all groups showed some degree of reduction in sealer-induced discoloration. In addition, in all groups, the largest difference in L was related to the difference in L0 and L2 (before bleaching and one week after bleaching again), and the lowest difference was related to the difference in L0 and L1. Also, the highest ΔE(T0,T1) belonged to the Endofill group, and this significantly differed from the AH26 group. AH26 showed the lowest value of ΔE(T0,T1), and after two weeks, the ΔE of all groups was higher than the clinically observable limit. The highest ΔE(T2,T0) among the groups belonged to the Endofill group. The ΔE(T2,T0) of AH26 and Endofill was significantly higher than AH Plus. Among all ΔE values, the AH Plus group had the lowest values.

Conclusion

Color change caused by Endofill and AH26 sealers showed a better response to internal bleaching than the AH Plus sealer.

## Introduction

The occurrence of discoloration resulting from using substances in endodontic therapy is a significant matter of concern in clinical practice [1]. The reason for these color changes is the remaining sealer in the pulp chamber [2]. The use of sealers with less discoloration potential and the complete removal and debridement of sealer and gutta-percha residues from the pulp chamber during restoration are solutions to prevent dentine discoloration caused by the sealer. However, for discolored teeth, the internal bleaching technique can be used to restore the natural color of the teeth. It is also possible to use veneers and laminate veneers, which is not a cost-effective treatment both economically and in terms of tooth tissue removal [3]. The sealer type and material used seem to impact the intensity and type of color change [1,4,5]. Several studies have been conducted on the potential of changing the color of sealers [4-6]. Partovi et al. have used computer analysis to investigate the color change caused by seven materials, including Endofill, ZnOE, Tubuliseal, AH26, gutta-percha, Cavisol, and apatite root sealer [5]. According to the results of this study, most of the alteration in color took place in the cervical third; the highest amount of color change was associated with Endofill, and the least was associated with distilled water.

Bleaching is a suitable treatment to reduce the color change caused by the sealer and has many advantages compared to veneers, including more preservation of the tooth structure and lower cost of treatment [6]. Internal bleaching is an effective and cost-efficient therapy method to address discoloration resulting from endodontic operations [7]. A potent oxidizing chemical is inserted into the pulp chamber in the bleaching process to change the pigmented material into colorless. The most common bleaching agents are hydrogen peroxide and its derivatives, namely, sodium perborate and carbamide peroxide. However, different concentrations, formulations, and combinations of the materials mentioned above and the use of heat or light have been proposed to enhance efficiency [7,8].

Recently, Ioannidis et al. have measured the intensity of color change induced by four sealers, Roth 811, AH26, GuttaFlow, and Epiphany SE, by means of a spectrophotometer and computer software. The computer analysis of color change was performed before sealer placement and one week, one month, three months, and six months after sealer placement, and in all groups, Roth 811 induced more intense color change [8]. It seems that the prognosis of bleaching treatment in reducing the color change caused by the sealer depends on some factors, including the type of sealer [7], the age of the person (eight and 10) [9], the duration of the color change, and the bleaching agent [10]. The inside/outside bleaching technique with 10% carbamide peroxide is the most effective and safest method of bleaching nonliving teeth [11]. In previous studies, the spectrophotometric method was not used to investigate the response of teeth to bleaching, and the comparison of the findings was based on the comparison with the color spectrum. The primary objective of the present research was to assess the impact of internal bleaching with 10% carbamide peroxide on discolored teeth with three common endodontic sealers.

## Materials and methods

Sampling

The Tabriz University of Medical Sciences Ethics Committee has approved this experimental research and has been given the ethical code 312. The current study was performed in 2015 at Tabriz Dental School's restorative department. A power and sample size software was used to determine the sample size. For this study, three groups were first considered, 12 samples in each group, and 36 samples were estimated. The inclusion criteria were healthy teeth without external or internal discoloration and young and fully mature teeth. Also, the exclusion criteria included root-treated teeth, decayed teeth, and a long time interval from the time of extraction to the beginning of the study.

Implementation method

This study involved selecting and gathering 36 anterior teeth from patients referred to the surgical department of Tabriz Dental Faculty. These teeth were extracted due to periodontal disease and could not be preserved. The extraction was carried out with the patient's consent. In all teeth, a cut was applied 1 mm below the cementoenamel junction (CEJ). A coronal access hole was not prepared to avoid other factors causing discoloration, such as temporary or permanent filling materials and microleakage caused by them, which cannot be easily evaluated. The pulp was taken out with an excavator, and the space of the pulp chamber was debrided with a barbed broach and Hedstrom number 30. The internal axial walls were gently abraded with Hedstrom numbers 60-80, and washing was done with normal saline. The smear layer was not removed. Sterile cotton balls dried the space of the pulp chamber. The removed dental crowns were stored in distilled water within a refrigerated environment throughout tooth collection.

After completing the collection of teeth and starting the study period, each tooth was placed in separate containers of distilled water and numbered. Then, they were divided into three groups: group 1 (Endofill, Herpo Produtos Dentários Ltda, Petrópolis, Brazil), group 2 (AH26, Tulsa Dental, Tulsa, OK), and group 3 (AH Plus, Dentsply DeTrey, Konstanz, Germany).

Following the manufacturer's directions, sealers were mixed and inserted into the pulp chamber via the cervical access hole that had been created previously. Sealer additions were removed with sterile cotton. Gutta-percha was not used to fill the pulp chamber space; instead, the cervical access cavity was covered with a thin layer of glass ionomer (GC Fuji IX GP, GC America, Alsip, IL). After one month of incubation [12] at 37°C, crown color was determined using a visible spectrophotometer (SpectroShade™ Micro, Medical High Technologies, Verona, Italy) before starting the bleaching treatment.

Bleaching process

After removing the glass ionomer layer, the sealer was removed from inside the pulp chamber, and the remaining sealers were removed with a pin dipped in chloroform solvent [13]. Ten percent carbamide peroxide gel (Opalescence 10% PF, Ultradent Products Inc., South Jordan, UT) was placed inside the pulp chamber [11]. The additions of bleaching agent were removed, and temporary restoration was done with dressing materials. The samples were kept in a humid environment at a temperature of 37° and were examined at the specified times. The colors were measured on the buccal surface and on the moist crown at three points. Then, the average values of these three points were calculated. The measurement time was T0 (before bleaching treatment), T1 (one week after bleaching treatment), and T2 (one week after re-bleaching treatment). L0, teeth volume before bleaching; L1, teeth volume one week after bleaching; and L2, teeth volume one week after bleaching, were determined. Also, ΔE(T0,T1) is the difference between the teeth's color before and one week after bleaching. ΔE(T2,T0) is the difference between the teeth's color before and after re-bleaching. ΔE(T2,T1) is the difference between the color of the teeth one week after the initial bleaching and one week after re-bleaching.

Statistical analysis

Statistical analysis was performed using the Statistical Package for Social Sciences (SPSS) software version 16 (IBM SPSS Statistics, Armonk, NY) with a significance level set at P<0.05. The data obtained from this study were analyzed using general linear model analysis and one-way analysis of variance (ANOVA). The homogeneity of the variance of the differences was confirmed by Mauchly's test of sphericity. Bonferroni's post hoc test was used for in-group and between-group pairwise comparisons to examine the components' principal impacts and interactions. 

## Results

The descriptive statistics related to L values and ΔE values in different stages of bleaching in different investigated groups are presented in Table 1 and Table 2, respectively. A comparison of the two groups with Bonferroni's prolonged test showed that there was a significant difference between Endofill and AH Plus and Endofill and AH26, but there was no significant difference between AH Plus and AH26. The ANOVA for the different stages of bleaching and index L showed that in all groups, the greatest difference was the difference in L0 and L2 (before bleaching and two weeks after bleaching) and the least difference was the difference in L0 and L1. In all groups, the difference between L1 and L2 was greater than the difference between L0 and L1 (P=0.06 versus P=0.002) (Table 1).

**Table 1:** Different stages of bleaching (mean±standard deviation) L0, teeth value before bleaching; L1, teeth value one week after bleaching; and L2, teeth value one week after re-bleaching

Group	N	L0	L1	L2	F	P-value
Endofill	12	69.2±51.95	71.1±55.99	77.2±77.96	0.634	0.0003
AH Plus	12	74.3±55.41	75.2±74.48	79.3±5.17	5.34	0.002
AH26	12	73.2±41.64	75.2±27.03	80.2±91.34	1.418	0.001

**Table 2:** ΔE values in different stages of tooth whitening in different groups (mean±standard deviation)

Group	N	ΔE(T0,T1)	ΔE(T2,T0)	ΔE(T2,T1)	F	P-value
Endofill	12	93.37±4.2	25.09±9.2	53.92±7.1	3.727	0.000
AH Plus	12	99.45±2.1	63.94±5.1	93.66±4.1	9.56	0.086
AH26	12	98.89±2.1	89.37±8.2	98.45±7.2	6.23	0.149

A comparison of the two groups with Bonferroni's prolonged test showed that the effect of whitening time (ΔE) was significant between the AH Plus and Endofill groups (P<0.005) but not significant between AH Plus and AH26 (P=0.09). The ANOVA for the various stages of bleaching and the ΔE index showed that after one week and two weeks of internal bleaching, all groups showed some degree of reduction of color change caused by the sealer. Only the Endofill group exhibited above the clinical limit (3.5<ΔE) after a week. The highest ΔE(T0,T1) belonged to the Endofill group, and this significantly differed from the AH26 group, but it was not meaningful to the AH Plus. AH26 showed the least value of ΔE(T0,T1). The difference in ΔE(T0,T1) between AH Plus and AH26 was insignificant. ΔE(T0,T1) obtained for AH26 and AH Plus were less visible than the clinical limit. After bleaching ΔE, all groups were higher than the clinical limit. The highest ΔE(T2,T0) among the groups was from the Endofill group. The ΔE(T2,T0) of AH26 and Endofill was significantly higher than AH Plus but was not significantly different. In all the values of ΔE, the AH Plus group had the lowest values (Table 2).

## Discussion

The current research examined the impact of internal bleaching with 10% carbamide peroxide on discolored teeth resulting from various types of sealers. This study showed that using AH26, AH Plus, and Endofill sealers for one month caused a significant color change inside the pulp chamber. The possible reasons for creating colored products can be because there is eugenol in the composition of Endofill sealer, and eugenol forms a bond with zinc oxide. Therefore, its residue is oxidized and darkens over time [12]. In addition, one of the other reasons for this darkness is that there is silver in the composition of AH26 sealer, which oxidizes over time and leads to the darkening of the tooth color [12]. In addition, AH26 silver-free sealer contains bismuth oxide, methenamine, and epoxy resin. Bismuth oxide is a yellow compound that is a pigment in cosmetics and paints [14]. AH Plus's manufacturer introduced the sealer as an alternative to the AH26 sealer, which does not have the severe color change problems of AH26, but the results of some studies have shown that the above-described sealer material also causes color change to a small extent [13]. One of the possible reasons for the color change is that in the sealer's chemical composition, there are some factors such as zirconium dioxide, bisphenol A, amantadine, and iron oxide [15], which may be effective in color change.

In the present research, 10% carbamide peroxide was used, the most effective and safest substance for the inside/outside bleaching method based on the results of previous studies [11,16-19]. Since bleaching agents can change or remove oxides due to their chemical composition, they are used to remove the color change caused by the sealer. The prognosis of bleaching treatment is mentioned in some studies, such as the person's age (eight and 10) and the duration of the sealer in the tooth [11], which seems to be related to the dentin tubules. Due to the inherent limitations of the human eye in perceiving small color variations and the subjective nature of interpreting visual color comparisons, color measurement tools are used to obtain accurate results. In the present study, the CIE L*a*b color measurement system was used, in which the L factor is related to enamel opacities and glossiness and is influenced by surface roughness, and is classified from black (zero) to white (100). The outcomes of this research indicate that the time and frequency of bleaching significantly affect the L and ΔE variables. In addition, this study also found that the L and E variables were significantly affected by the influence of sealer type and the inverse effect of time and sealer type.

Based on the results of the present study, it can be argued that the success of the internal bleaching treatment in removing the color change caused by the sealer depends on the contact of the bleaching agent with the remaining sealer and colored products in the dentin. For this reason, one of the reasons for the less response of AH26 and AH Plus to bleaching treatment in this study (after one week of bleaching) and AH26 in previous studies may be the greater penetration of epoxy resins into the dentine tubules [15] and the lack of contact of the bleaching agent with all the sealer in the dentin. On the other hand, the Endofill sealer, which is a ZnOE-based sealer, may have come into contact with the bleaching agent during the first week of bleaching and become whiter due to less penetration in the tubules than the resin sealers. Also, the tendency of the recurrence of color changes that have been reported in cases such as in AH26 [10] can be justified by this hypothesis. After one and two weeks of internal bleaching, all groups showed some degree of reduction in sealer-induced discoloration, which is consistent with the findings of the study by Gürel et al. (2016) [11] and also the research results of van der Burgt and Plasschaert (1986) [9].

Based on the results of the present study, AH26 showed the lowest ΔE value after one week, which is consistent with previous studies. In addition, the difference of ΔE(T0,T1) between AH Plus and AH26 was insignificant. Also, the ΔE(T0,T1) obtained for AH26 and AH Plus was lower than the clinically observable limit, which was different from other studies, and this is probably due to the different bleaching agents. In the study by Gürel et al. (2016) [11], 35% hydrogen peroxide was used, and the ΔE obtained after one week of bleaching a discolored tooth with AH26 sealer was reported to be 34.6. However, in the present study, 10% carbamide peroxide was used, and the ΔE obtained after one week of bleaching the discolored tooth with AH26 sealer was equal to 2.98. According to the results of the present study, after two weeks, the ΔE value of all groups was higher than the clinically visible limit, while in other studies, the clinically visible ΔE value was obtained after one week. The possible reason for this difference of one week may be related to the auxiliary bleaching agent. The study by Feiz et al. (2014) [19] reported that significant changes in ΔE were obtained in all three bleaching agents after two weeks. It seems that carbamide peroxide needs more time than hydrogen peroxide to induce clinical effects. The study by Gürel et al. (2016) [11] showed that Pulpispad has the most color change among the four groups, and after bleaching, EndoREZ showed a better response to internal bleaching treatment than the other three sealers, which is consistent with the results of the present study. It is necessary to explain that the type of sealers tested and the type of bleaching material in the research by Gürel et al. (2016) [11] are different from the present research. Due to the existence of different studies in this field and its importance due to its impact on the discussion of beauty, more studies in this field are required.

Limitations of the study

This study possesses certain limitations. Firstly, it is important to note that the study was conducted in vitro, and thus, if it was conducted in vivo, the results would likely be more valid and valuable. Secondly, there are limitations in terms of the variety of sealers used in the study, as well as the relatively small number of sealers included. This limitation may impact the generalizability of the findings. Furthermore, the duration of the study is worth considering, as the teeth were only exposed to the sealers for approximately one month. This timeframe may not be sufficient to observe color changes and assess other relevant changes. A longer waiting period before bleaching could yield more comprehensive results. In light of these limitations, we propose that future research explore the use of a wider range of materials for bleaching within the canal, as well as investigate alternative methods such as external bleaching.

## Conclusions

In summary, the findings obtained from the spectrophotometer measurements indicated a significant enhancement in coloration across all experimental groups. The observed color alteration resulting from using the Endofill and AH26 sealers exhibited a more favorable reaction to internal bleaching than the AH Plus sealer. The current investigation also revealed that before the permanent restoration placement, it is imperative to meticulously eliminate the sealer within the tooth's pulp chamber after root treatment.
